# Context-dependent selection sculpts mutational landscapes in the normal lung

**DOI:** 10.21203/rs.3.rs-10195584/v1

**Published:** 2026-07-02

**Authors:** Edward J. Evans, Ferriol Calvet, Fabio Marongiu, Faiz Jabbar, Elan Z. Eisenmesser, William Hill, Clare Weeden, Oriol Pich, Moumita Ghosh, Robert L. Keith, York E. Miller, Daniel T. Merrick, Clara I. Troccoli, Andrew Rowan, Takahiro Karasaki, Selvaraju Veeriah, David A. Moore, Mihaela Angelova, Olivia Lucas, Charlotte Grieco, Lydia Y. Liu, Cristina Naceur-Lombardelli, TRACERx Consortium, PEACE Consortium, Mariam Jamal-Hanjani, Nuria Lopez-Bigas, Emilia L. Lim, Charles Swanton, James DeGregori

**Affiliations:** 1. Departments of Biochemistry and Molecular Genetics, Immunology and Microbiology, and Medicine, University of Colorado Anschutz Medical Campus, Aurora, CO, USA; 2. Institute for Research in Biomedicine (IRB Barcelona), The Barcelona Institute of Science and Technology, Baldiri Reixac 10, 08028 Barcelona, Spain; 3. Department of Biomedical Sciences, University of Cagliari, Italy; 4. Cancer Evolution and Genome Instability Laboratory, The Francis Crick Institute, London, UK; 5. Cancer Research UK Lung Cancer Centre of Excellence, UCL Cancer Institute, London, UK; 6. Department of Radiology, King’s College Hospital, London, UK; 7. Cancer Research UK Manchester Institute, The University of Manchester, Manchester, UK; 8. Walter and Eliza Hall Institute of Medical Research, Parkville, VIC, Australia; 9. Department of Medical Biology, University of Melbourne, Melbourne, VIC, Australia; 10. Department of Medicine, Division of Pulmonary, Allergy, and Critical Care Medicine / Pulmonary Sciences and Critical Care Medicine, University of Colorado Anschutz Medical Campus, Aurora, CO, USA; 11. Rocky Mountain Regional Veteran’s Administration Medical Center, Aurora, CO, USA; 12. Department of Pathology, University of Colorado Anschutz Medical Campus, Aurora, CO, USA; 13. Present address: Precision Therapeutics, Philadelphia, PA, USA; 14. Department of Thoracic Surgery, Graduate School of Medicine, The University of Tokyo, Tokyo, Japan; 15. Department of Medical Science and Innovation, SiRIUS Institute of Medical Research, Tohoku University, Sendai, Japan; 16. Department of Cellular Pathology, University College London Hospitals; 17. Department of Medical Oncology, University College London Hospitals, London, UK; 18. Computational Cancer Genomics Research Group, University College London Cancer Institute, London, UK; 19. Cancer Metastasis Laboratory, University College London Cancer Institute, London, UK; 20. Centro de Investigación Biomédica en Red en Cáncer (CIBERONC), Instituto de Salud Carlos III, Madrid, Spain; 21. Department of Medicine and Life Sciences, Universitat Pompeu Fabra, Barcelona, Spain; 22. Institució Catalana de Recerca i Estudis Avançats (ICREA), Barcelona, Spain; 23. Department of Biochemistry and Molecular Biology, The University of British Columbia, Canada; 24. Edwin SH Leong Centre for Healthy Aging, The University of British Columbia, Canada

## Abstract

Just as organismal evolution is driven by environmental changes, somatic evolution is similarly driven by changes in tissue environments, whether caused by the normal process of aging, by lifestyle choices, or by extrinsic exposures. We present deep mutational profiling using duplex sequencing of over 390 samples of non-malignant lung tissue from 202 subjects, showing how aging, smoking and different anti-cancer therapies are associated with selection for particular mutations in normal lung tissue. Mutations within known cancer driver genes exhibit selection in the normal tissue that differs from that for lung cancers, with a substantially expanded spectrum of sites under selection. Multiregional profiling of mutational landscapes reveals convergent evolution within each individual’s lung. Understanding the forces controlling clonal selection as we age and due to lifetime exposures could be critical for controlling multiple diseases of old age.

## INTRODUCTION:

The process of natural selection acts on heritable phenotypic diversity in a population to select for individuals that are better adapted to their current environment. Together with genetic drift, the forces of mutation (generating diversity) and selection (picking winners) drive evolution. These rules hold true not only for organismal populations but also for somatic evolution within our bodies and those of all animals. While the prior focus of cancer research has been on the occurrence of mutations, and how carcinogenic contexts increase the frequency of these mutations, there is growing appreciation for the evolutionary dynamics that can act as promoting events to influence the selection and expansion of specific mutant clones^1^. We have previously postulated that oncogenesis is fundamentally adaptive in nature, and that environmental changes (e.g. aging, carcinogenic exposures, inflammation) can shift selective pressures in tissues, promoting increased positive selection for some variants and negative selection for others^2^.

Moreover, while the accumulation of mutations is typically considered as inherently detrimental, recent studies show that phenotypically normal tissues across the body (e.g. esophagus, skin, colon, and blood) are riddled with known cancer-associated mutations that clonally expand with age or insult^3–14^. Yet only rarely do these clones progress to malignancy^1,15,16^. Moreover, much less is known about the extent and determinants of clonal expansion in the normal human lung^17,18^. While most earlier studies identified mutation-associated clonal expansions using sequencing methods with limits of detection of >1%, more recent studies in the bladder epithelium, buccal epithelium, blood, and other tissues using duplex sequencing (DuplexSeq; with limits of detection <10^−4^) have enabled deeper insight into clonal architecture and influences of factors ranging from sex to cigarette smoking to anti-cancer treatment^19–22^. These studies indicate that our tissues have evolved multiple barriers against malignant evolution, even in the presence of substantial mutational burden^2,16,23^. Still, it remains unclear what the significance of clones bearing specific mutations is, and what their selective clonal expansion implies in terms of tissue health, cancer risk, and the pathogenesis of other diseases.

Cigarette smoke contains numerous mutagenic chemicals and, importantly, can profoundly damage lung tissue, leading to chronic inflammation, barrier disruption, reduced progenitor function, and altered immune and stromal interactions^17,24,25^. These smoking-induced alterations could reshape the tissue environment in ways that promote positive selection for clones with adaptive mutations. Smoking has also been shown to alter selection for mutations in specific genes in the esophagus^13,14^, bladder^21^, and as a driver of clonal hematopoiesis^26–28^. Notably, sequencing of single cell-derived clones from bronchial epithelium revealed the striking influence of smoking on mutation number and patterns, and the high frequency of cancer-associated mutations^17^. Similarly, air pollution increases cancer risk by contributing to the formation of an inflammed tissue microenvironment that enables the expansion of pre-existing mutant clones^29^. By analogy, smoking and other tissue insults such as anti-cancer therapies are expected to influence clonal dynamics in the lung; however, how they do so, particularly at the level of specific mutations, remains largely unknown.

We can thus appreciate that distinct tissue contexts, whether altered by smoking, therapies, pollution or age, differentially shape which variants gain or lose selective advantage. Under this model, the mutational landscape of normal tissue is not expected to simply recapitulate that of overt cancer. Instead, some mutations may be favored in normal tissue because they confer a competitive advantage in a particular ecological setting without necessarily representing the canonical set of alterations that dominate advanced tumors. Defining these selective landscapes in normal tissue is therefore essential for understanding how age and exposure shape early somatic evolution before malignant transformation.

To address these questions in normal human lungs, we analyzed histologically normal tissue samples from three patient cohorts, leveraging DuplexSeq technology with a targeted ~50 kb panel across mutational hotspots in 27 cancer-associated genes, to quantify mutation burden and clonal expansions at high resolution. Here, we demonstrate that smoking and therapies act not only as mutagens but as promoters of positive selection for particular mutations. Recurrent mutational patterns emerge within an individual, indicating reproducible selective forces. Moreover, the mutational landscapes observed in normal lungs only partially overlap with those of lung cancers, with a broader spectrum of sites under selection in normal tissue. Notably, smoking shifts somatic evolution toward mutational landscapes that more closely resemble those observed in lung cancer. Thus, distinct contexts, from aging to smoking to therapies, appear to favor divergent evolutionary trajectories in normal lung.

## RESULTS:

### Aging and smoking increases mutation burden and mutational signatures in histologically normal tissue

**Figure 1.**
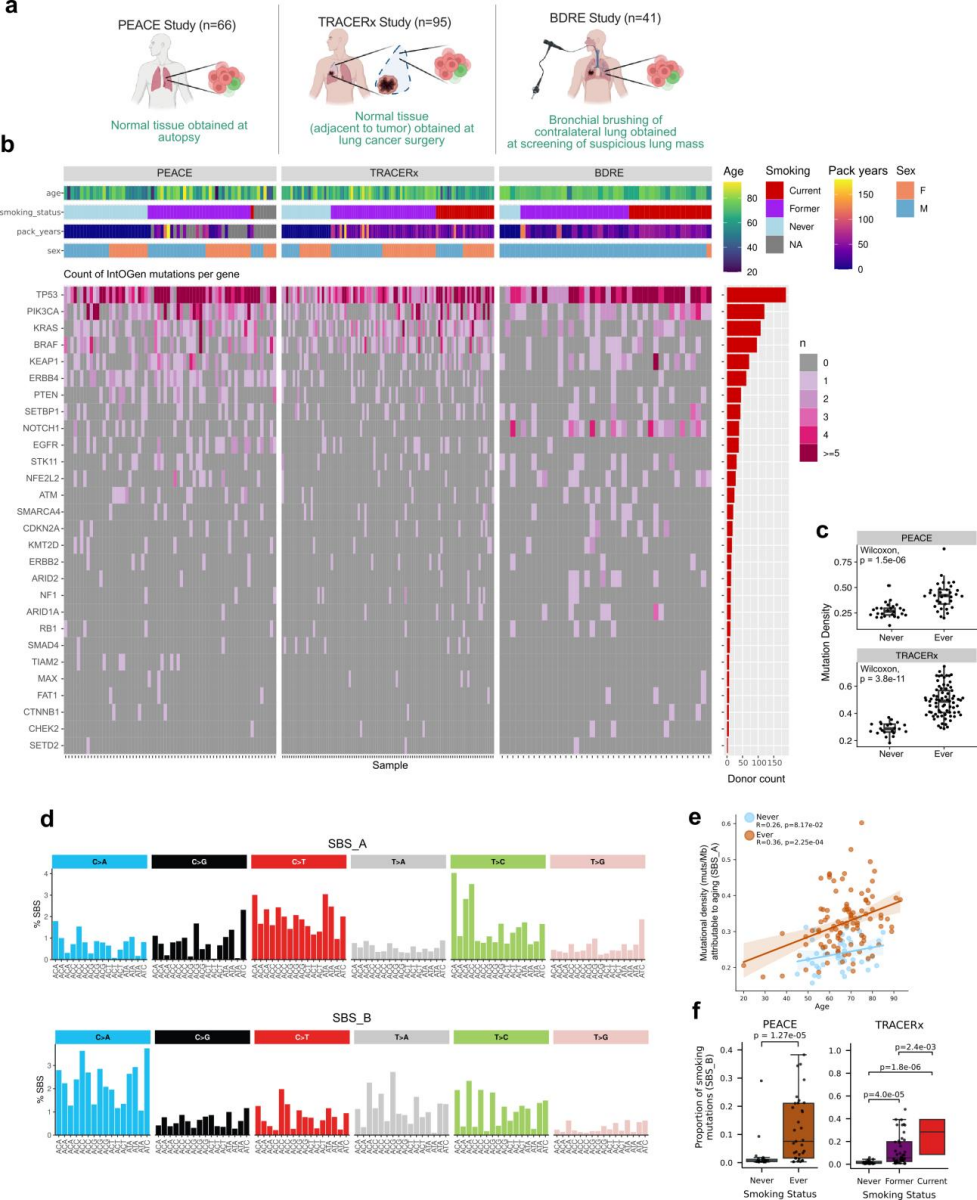
Smoking enhances mutation prevalence and smoking signatures in cancer-free, histologically normal lung. **a**, Depiction of the acquisition of lung samples in the PEACE, TRACERx and BDRE cohorts. **b**, Distribution of participant meta-data (age, smoking status, number of pack years, and sex), mutation density distinguishing for the proportion of observed mutations in IntOGen, and a heatmap of the prevalence of IntOGen mutations by gene in the samples across the three cohorts. **c**, Box and whisker plot to display the Wilcoxon test comparisons of the mutation density vs smoking status for PEACE and TRACERx cohorts. **d**, Trinucleotide mutation spectra of aging- (SBS_A, top) and smoking-associated (SBS_B, bottom) mutational signatures, extracted using Hierarchical Dirichlet Process with the mutations in the PEACE and TRACERx cohorts. **e**, Scatter plot showing the Pearson correlations between mutation density attributable to aging and the age of PEACE and TRACERx donors across smoking groups. Coefficients and p-value for the relationships are indicated. **f**, Box and whisker plots to display the Wilcoxon test comparisons of the proportion of mutations attributed to the generated SBS_B smoking-associated mutational signature across smoking status in the PEACE (left) and TRACERx (right) cohorts.

To investigate how smoking influences somatic mutational landscapes in non-malignant lung tissue, we performed DuplexSeq using a ~50 kb cancer-focused panel (**Extended Data Table 1**) across histologically normal lung samples from three independent cohorts (Fig. 1a). TRACERx (*Tracking Non-Small-Cell Lung Cancer Evolution through Therapy*; NCT01888601), is a prospective longitudinal study of non-small cell lung cancer (NSCLC), providing histologically normal lung tissue at least 2 cm away from tumor at the time of primary surgery^30,31^. PEACE (*Posthumous Evaluation of Advanced Cancer Environment*; NCT03004755), is a UK pan-cancer research autopsy program, providing histologically normal background lung tissue from individuals with cancer^32^. Samples of normal lung from PEACE and TRACERx contain an estimated 26% epithelial cell content^33^, whereas the epithelial content of brushings is typically >89%^29^ (see Methods). To enrich sampling of epithelia and provide insight into early somatic evolution at the most common sites of origin for NSCLC, we utilized the Biomarkers of Dysplastic Respiratory Epithelium (BDRE) cohort. Individuals in the BDRE cohort were referred for endobronchial brushing of peripheral lung nodules following screening of high-risk current and former smokers via low dose computed tomography (LDCT). In addition to the indicated brushing obtained at the site of a suspected lesion (involved site), these individuals also consented to a research brushing at a contralateral site, in a different lobe that displayed no evident pathology. Roughly half of the BDRE subjects analyzed were diagnosed with lung cancer, while the others remained radiologically cancer free with at least two years of follow up with serial LDCTs.

Across PEACE and TRACERx, participants spanned a broad age range, showed a balanced distribution of sexes, and encompassed diverse smoking histories (Fig. 1B). BDRE subjects were mostly male and mostly smokers, reflecting the veteran population recommended for LDCT. See **Extended Data Fig. 1** for sample selection criteria, **Extended Data Table 2** for patient characteristics, and **Extended Data Table 3** for confounding factor associations with cigarette smoking histories. We will refer to individuals based on smoking history as those who have never smoked (never-smokers) or ever smoked (ever-smokers), including former-smokers and current-smokers, at the time of sampling.

Focusing on PEACE and TRACERx given sufficient representation of never-smokers, individuals with a history of smoking exhibited a significant increase in overall mutation density compared to never-smokers (Fig. 1b,c), consistent with previous studies^17,20^. It is important to emphasize that the detection of a mutation will reflect some combination of mutation rate, selection, and drift^34^. IntOGen-annotated mutations, a compendium of cancer driver mutations^35^, were detected across many samples, typically with multiple driver genes in each sample, although the affected genes varied substantially between samples (Fig. 1b). Roughly 5–10% of mutations detected across cohorts were IntOGen-annotated (**Extended Data Fig. 2**). Smoking-stratified analyses of age-associated mutation burden revealed a positive relationship between age and total mutation density in never-smokers (**Extended Data Fig. 3**, left). In ever-smokers, this age dependence was attenuated or lost, likely obscured by the high numbers of smoking-associated mutations. Mutation densities were largely similar across sexes (**Extended Data Fig. 3**, right).

Two independent *de novo* signature extraction methods identified two dominant mutational patterns that we refer to as SBS_A and SBS_B in the combined PEACE and TRACERx cohorts (Fig. 1d, Methods). SBS_A can be mostly reconstructed with COSMIC SBS5 (clock-like endogenous mutational processes) and SBS_B with a combination of SBS4 (tobacco-associated mutagenesis) and SBS5 (**Extended Data Fig. 4a, b**). The extracted signatures were used for downstream analysis as these capture the different exposures of the donors with higher accuracy compared with the COSMIC deconstruction of the mutational patterns. This is likely attributable to a low resolution for detecting mutational signatures rather than the discovery of new mutational processes. Despite this limitation, the mutational landscape of normal lung in our samples is shaped mainly by these two processes.

Mutation density attributed to SBS_A increased significantly with age in ever-smokers (Fig. 1e), contrasting with the total mutation counts. The proportion of mutations attributed to SBS_B varied significantly with smoking status (Fig. 1f). In PEACE, SBS_B contribution was higher in ever-smokers than in never-smokers, whereas in TRACERx all pairwise comparisons between never-smokers, former, and current smokers were significant, consistent with a stepwise increase in SBS_B attribution across exposure groups. These findings indicate that tobacco exposure leaves a persistent and exposure-dependent mutational imprint in histologically normal lung tissue, consistent with previous results^17,20^.

Across the combined PEACE and TRACERx cohorts, smoking-signature mutation density increased with cumulative smoking exposure (pack-years), albeit with substantial inter-individual variability and only when never-smokers were included in the analysis (**Extended Data Fig. 4c,d**). Aging-signature mutation density also increased with pack-years (only when never-smokers were included); an increase of SBS5-like mutations with smoking has been reported^17,36^, but this association should be interpreted cautiously given the likely correlation between pack-years and age.

Together, these data provide evidence that smoking is associated with increased mutation density in histologically normal human lung and with a detectable tobacco-related mutational component.

### Smoking alters selection and subsequent clonal patterns in lung tissue

**Figure 2.**
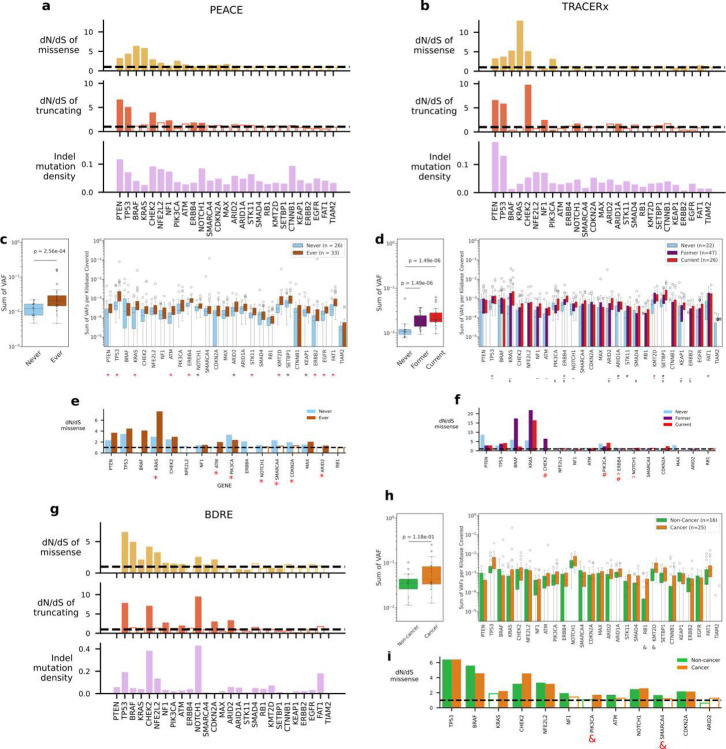
Positive selection influenced by smoking history drives gene-specific clonal expansions in normal lung tissue. **a** and **b**, positive selection metrics by gene for the PEACE (**a**) and TRACERx (**b**) cohorts, showing histograms of dN/dS calculations for missense & truncating mutations and the mutation density of indels. Except for indel mutation density plots, filled in shapes indicate statistical significance. **c** and **d**, Box and whisker plots of the sums of VAFs of protein-affecting mutations by smoking status across the sequencing panel (left) and by gene within the panel (right) for the PEACE (**c**) and TRACERx (**d**) cohorts. Black and red symbols indicate the unadjusted and adjusted, respectively, p-values < 0.05 for comparisons in cohorts. The #, ^, and $ symbols indicate never-former, never-current, and former-current comparisons, respectively. **e** and **f**, barplots of dN/dS calculations for missense mutations in genes under selection stratified by smoking status in the PEACE (**e**) and TRACERx (**f**) cohorts. Filled in bars indicate significant selection, and the *, #, ^, and $ symbols indicate significant differences between groups of donors based on their smoking history. **g**, Positive selection metrics by gene for BDRE, showing histograms of dN/dS calculations for missense and truncating mutations, and the mutation density of indels. In all panels except indel mutation density, filled in shapes indicate statistical significance. **h**, For BDRE, box and whisker plots of the sums of VAFs of protein-affecting mutations by cancer diagnosis across the sequencing panel (left) and by gene within the panel (right). **i**, Bar plots of dN/dS calculations of missense mutations in genes under selection stratified by cancer diagnosis (BDRE). For **h** and **i**, black and red symbols indicate that unadjusted and adjusted, respectively, p-values < 0.05 (& symbol) for comparisons based on cancer diagnosis. For c, d, and h the t-test or Mann-Whitney U Test was conducted depending on the normality of the data. Differences between groups in e, f and i were computed by comparing the proportions of excess driver mutations using Fisher’s exact test. Multiple comparisons were accounted for using Benjamini-Hochberg correction where applicable.

We next examined whether somatic mutations exhibit evidence of positive selection and whether smoking modifies the strength of such selection. Gene-level selection was quantified using dN/dS for missense and truncating mutations, together with complementary analyses of indel density, three-dimensional clustering^37^, and functional impact bias^38^ (likelihood that a mutation alters protein function) across genes in the PEACE and TRACERx cohorts (Fig. 2a,b; **Extended Data Figure 5**). The strength of selection can be estimated using dN/dS^39^. We employed omega, a method that accounts for the differences in sequencing depth per site which are known to affect the computation of the dN/dS estimates when working with DuplexSeq data^21^. dN/dS ratios significantly greater than or less than 1 indicate positive or negative selection, respectively. The mutation density of insertions and deletions (indels) was used as a proxy of selection for these types of mutations.

Across both cohorts, multiple genes displayed dN/dS ratios significantly exceeding 1, consistent with positive selection in normal lung tissue (Fig. 2a,b). Selection followed canonical functional patterns where missense mutations were enriched in genes with gain-of-function mutations common in cancers such as *KRAS*, *BRAF* and *PIK3CA*, whereas truncating mutations were preferentially selected in genes with loss-of-function mutations in cancers such as *TP53* and *PTEN*. In contrast, *TIAM2*, which is not designated as an IntOGen cancer driver gene^35^ and is used as an internal reference gene, did not exhibit evidence of positive selection across mutation types. Indel mutation density mirrored the pattern observed for truncating mutations, with enrichment in genes typically associated with loss-of-function mutations in cancers. Genes with elevated dN/dS also demonstrated significant three-dimensional clustering of missense mutations and enrichment for predicted high-impact variants, supporting non-neutral evolutionary processes in normal lung epithelium (**Extended Data Figure 5**). Several well-known NSCLC driver genes did not show significant selection at the gene-level, but mutations in specific sites were significantly selected for (**Extended Data Table 4**). For example, the cancer hotspot mutations *EGFR* L858R and *ERBB2* S310F mutations exhibited significant site selection.

We next evaluated the sum of the variant allele frequency (VAF) of protein-altering mutations (per sample and per gene per sample) as an aggregate measure of mutational representation that integrates both mutagenesis and clonal selection. This calculation allows us to determine how smoking histories alter the fraction of tissue occupied by mutation-driven clones (relative to the reference gene *TIAM2*). For heterozygous mutations (assuming that each mutation is in a different cell), doubling the sum of VAF should reflect the proportion of cells in a lung sample with the mutation. Across the panel, individuals with a smoking history exhibited higher sum of VAF values in both cohorts (Fig. 2c,d, left panel). At the gene level, this effect was heterogeneous, with larger smoking-associated differences observed for a subset of genes (Fig. 2c,d, right panel). Conversely, never-smokers did not have a higher sum of VAF for any gene, highlighting how smoking increases the proportion of mutated cells whether through mutation occurrence, expansion, or both. When comparing to *TIAM2* in the PEACE and TRACERx cohorts, the sum of VAF for most genes was significantly greater for both never-smokers and ever-smokers (**Extended Data Table 5**), likely reflecting increased selection acting on these genes across a lifetime.

To directly assess the impact of smoking on selection strength, we stratified dN/dS analyses by smoking status (Fig. 2e,f). Several genes exhibited smoking-associated differences, indicating that representation of protein-altering mutations among expanded higher-VAF clones differs across smoking groups. These selection patterns were gene- and cohort-specific. We observed consistent positive selection for genes such as *PTEN*, *TP53*, and *KRAS* in lung samples from never- and ever-smokers. Nonetheless, for PEACE, we observed significantly greater selection on *KRAS*, *ATM*, and *ARID2* mutations in ever-smokers but greater selection for *PIK3CA*, *NOTCH1*, *SMARCA4*, and *CDKN2A* in samples from never-smoker individuals. For TRACERx, we observed the strongest selection on *KRAS* and *BRAF* genes, with significant selection in all three smoking categories. We note that potentially confounding variables (age, sex and treatments) are largely similar within a cohort for individuals of different smoking histories (**Extended Data Table 3**).

Together, these analyses demonstrate that histologically normal lung tissue is subject to substantial positive selection on cancer driver genes, and that smoking modifies the strength of selection in specific genes, extending beyond its effect on mutational input.

While lacking in never-smokers, brushings from BDRE were enriched for epithelial cells, and around half of BDRE subjects were cancer-free. Mutational patterns were similar for BDRE contralateral samples (from the lobe with no evident pathology) compared to PEACE and TRACERx normal lung samples, and we observed similar mutation prevalence and selection for *TP53*, *KRAS*, *CHEK2*, and *BRAF* (Fig. 1a, 2g). Still, *NOTCH1* was prevalently observed, with signals of selection consistent with its known loss-of-function role, while *PTEN wa*s not under significant selection in these samples, which is notably different from PEACE and TRACERx. This difference across cohorts could reflect either the location of the samples (peripheral lung for BDRE) or the manner of sampling (brushings). Calculation of sum of VAF for genes in the panel revealed increases in genes associated with cancer relative to the reference *TIAM2* (which exhibited only 3 protein-altering mutations across 2 samples) (**Extended Data Fig. 5d**). Increased sum of VAF in current-smokers and former-smokers (relative to never-smokers) was evident for all genes combined (left) and for multiple individual genes, despite the paucity of smokers. By dN/dS, selection was stronger on multiple genes in current-smokers and former-smokers relative to never-smokers (**Extended Data Fig. 5e**).

At the site of the suspected malignancy, we detected cancer-associated mutations at higher VAFs in those who were diagnosed with cancer at the location of the brushing such as the *KRAS* G12D in participant #67 observed at ~10% VAF (**Extended Data Fig. 6**). We did not observe any variants that were >1% VAF at the involved site that were also detected at the matched contralateral site (see Methods), indicating that mutations detected at the contralateral site are unlikely to come from the lung cancer in the same individual (e.g. from circulating tumor DNA). We were able to compare variant patterns at the contralateral site based on the presence or absence of cancer at the involved site. Interestingly, we did not observe significant sum of VAF differences depending on whether cancer was diagnosed at the involved site (Fig. 2h) and only minor selection differences in *PIK3CA* and *SMARCA4* (Fig. 2i). In all, we conclude that mutational landscapes were not meaningfully different between the cancer and non-cancer groups in BDRE.

### Anti-cancer therapy exposure impacts the mutational landscape in the lung

**Figure 3.**
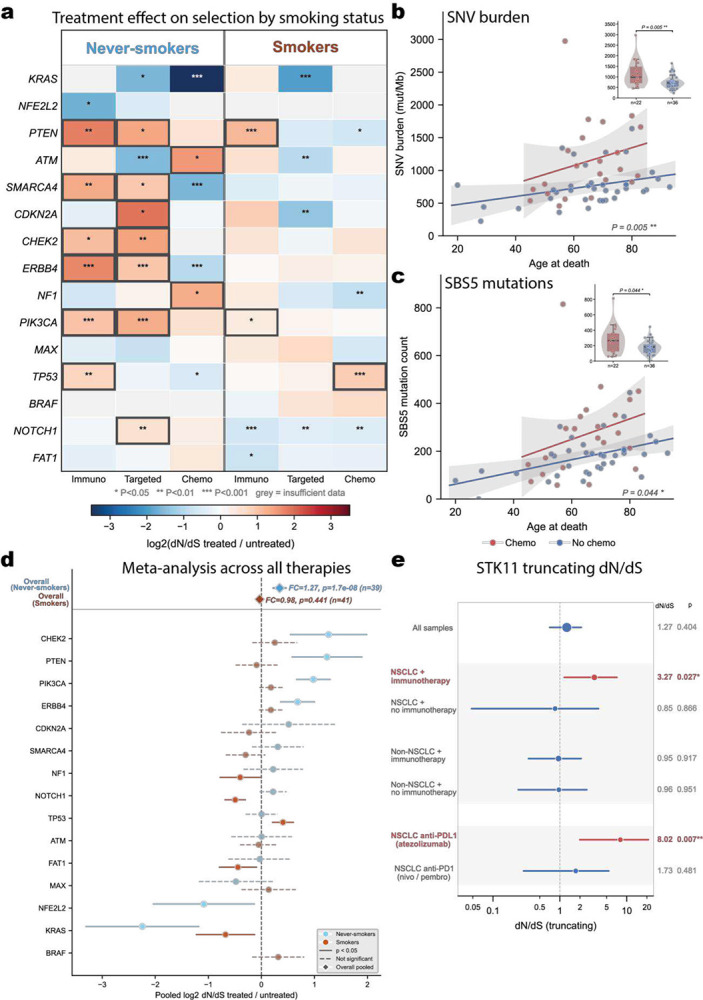
Therapy can impact mutation-driven clonal selection in the normal lung. **a**, Heatmap showing the log2 fold change of dN/dS (treated / untreated) for missense mutations across the 15 cohort-significant genes, stratified by smoking status (left: never-smokers; right: smokers) and therapy type (immunotherapy, targeted therapy, chemotherapy). Cells are colored by the RdBu diverging scale: red indicates higher dN/dS in treated patients, blue indicates higher dN/dS in untreated patients, and grey denotes insufficient data. Bold borders mark significant positive effects. Asterisks indicate Poisson likelihood ratio test significance (*P<0.05, **P<0.01, ***P<0.001). **b**, SNV mutational burden (mutations per Mb) as a function of age for chemotherapy-treated (red) and untreated (blue) patients, with linear regression lines and 95% confidence intervals. Inset: violin and box plots of the same data (Mann-Whitney U test). **c**, As in b, for SBS5 (clock-like) mutation counts attributed by SigProfilerAssignment. **d**, Fixed-effects inverse-variance-weighted meta-analysis of the log2(dN/dS treated / untreated) pooled across immunotherapy, chemotherapy and targeted therapy, shown separately for never-smokers (blue) and smokers (brown). **e**, STK11 loss-of-function selection (truncating dN/dS) stratified by tumor type, immunotherapy exposure and drug class. The NSCLC immunotherapy, no-immunotherapy, and anti-PDL1 subgroups were composed almost entirely of ever-smokers (9/10, 6/6 and 5/5, respectively), and all five patients who received anti-PDL1 therapy had NSCLC.

Prior DuplexSeq mutational analyses across 16 organs from 22 individuals in PEACE revealed positive selection on specific genes dependent on the therapies received^20^. For example, Pich et al observed increased selection for mutations in *TP53*, *CHEK2* and *PPM1D* in normal tissues including the lungs of patients treated with immunotherapy or chemotherapy. Given our larger cohort of individuals specifically analysed for mutations in normal lung tissue (66 individuals), we determined how different therapies impacted selection on specific genes in the lungs, comparing those receiving a particular class of therapy to all others in the PEACE cohort. Treatment exposure was broadly associated with elevated dN/dS ratios across immunotherapy, chemotherapy, and targeted therapy, with enrichment of missense mutations in multiple genes, with notable differences for the different therapies (Fig. 3a; **Extended Data Fig. 7**). Consideration of smoking history was critical. For immunotherapy, we observed increased selection for *PTEN, ERBB4, SMARCA4, CHEK2, PIK3CA* and *TP53*, and decreased selection for *NFE2L2*, in treated never-smokers (Fig. 3a). Only increased selection for *PTEN* and *PIK3CA* and decreased selection for *NOTCH1* and *FAT1* were evident in the ever-smokers. For some genes, such as *KRAS*, differences in selection (+/− immunotherapy) observed in the full cohort (**Extended Data Fig. 7**) could be fully explained by smoking status (Fig. 3a; **Extended Data Fig. 7; Extended Data Table 6**).

Strikingly different alterations in gene-specific selection were evident with chemotherapy. We observed increased selection for mutations in *ATM* and *NF1*, and decreased selection for *TP53, ERBB4, SMARCA4* and *KRAS* in treated never-smokers. In contrast, increased selection for *TP53* mutations and decreased selection for *NOTCH1, PTEN* and *NF1* mutations were evident in treated ever-smokers (Fig. 3a; **Extended Data Fig. 7**). For patients receiving targeted therapies, we observed increased selection acting on multiple genes including *CHEK2, ERBB4, PIK3CA* and *NOTCH1* only in never-smokers, with decreased selection acting on *KRAS* and *ATM* in both never and ever-smokers. Finally, while altered selection for mutations in *PTEN, PIK3CA* (both up), *MAX* and *SMARCA4* (both down) was evident in patients receiving hormonal therapy (**Extended Data Fig. 7**), we did not have sufficient sample numbers to stratify by smoking status. Thus, we observe clear influences of different therapies on selection for mutations in different genes, with results substantially impacted by smoking histories.

Beyond its effects on gene-specific selection, chemotherapy was also associated with an elevated overall mutational burden in normal lung tissue. Chemotherapy-treated patients had significantly higher SNV burden than untreated patients (Mann-Whitney P = 0.005; Fig. 3b), an association that remained significant after adjusting for smoking status and age in a multivariate linear regression (β = +298 mut/Mb, P = 0.010), although this effect was driven primarily by ever-smokers (stratified Mann-Whitney: smokers P = 0.034, never-smokers P = 0.441). Mutational signature decomposition attributed the excess mutations predominantly to SBS5, a clock-like signature associated with ageing: SBS5 mutation counts were significantly higher in chemotherapy-treated patients (P = 0.044; Fig. 3c). We did not observe significant changes in mutation burden for lung samples from patients treated with immunotherapy or targeted therapy (**Extended Data Fig. 8**).

When we pooled data for patients treated with immunotherapy, chemotherapy, and targeted therapy, we saw fold changes in dN/dS of up to 2.4-fold in treated versus untreated individuals (Fig. 3d). Interestingly, the treatment effect on selection was almost entirely confined to never-smokers. A fixed-effects meta-analysis pooling all gene-treatment combinations revealed that never-smokers who received therapy had a 27% higher dN/dS than untreated never-smokers (pooled log2 FC = +0.345, p=1.7×10^−8^), whereas ever-smokers showed no significant difference between treated and untreated groups (log2 FC = −0.032, p=0.44) (Fig. 3d). This pattern was consistent across immunotherapy (Wilcoxon p = 0.046), chemotherapy (p=0.103), and targeted therapy (p=0.153), and was driven by *CHEK2*, *PTEN*, *PIK3CA* and *ERBB4*, which showed significant treatment-associated increases in selection exclusively in never-smokers, whilst only *TP53* showed treatment-associated increases in selection in ever-smokers. *KRAS* mutations were under stronger selection in the untreated group for both never and ever-smokers. Overall, these analyses of the PEACE cohort reveal substantial influences of different therapies on gene-specific mutational selection in the normal lung, particularly in never-smokers.

Finally, we investigated whether immunotherapy could drive selection for specific loss-of-function events in *STK11* in normal lung tissue, given that mutations of this gene are an established mechanism of immune evasion in NSCLC, promoting resistance to anti-PDL-1 therapy^40^. *STK11* truncating mutations (nonsense and essential splice) showed no evidence of positive selection across the full cohort (dN/dS = 1.27, P = 0.40), but stratification by tumor type and treatment revealed anti-PDL1 therapy (atezolizumab) in NSCLC patients was associated with strong positive selection for *STK11* truncating mutations in normal lung (dN/dS=8.02, P=0.007; Fig. 3e), a signal absent in the normal lung of NSCLC patients not treated with immunotherapy, non-NSCLC patients regardless of treatment, and patients with NSCLC treated with anti-PD1 agents. Our findings suggest that immune-mediated selection pressure via anti-PDL1 immunotherapy favours clones with immune-evasive mutations in normal tissue.

### Site specific mutational analysis highlights the influence of smoking on selection for cancer-associated mutations

**Figure 4.**
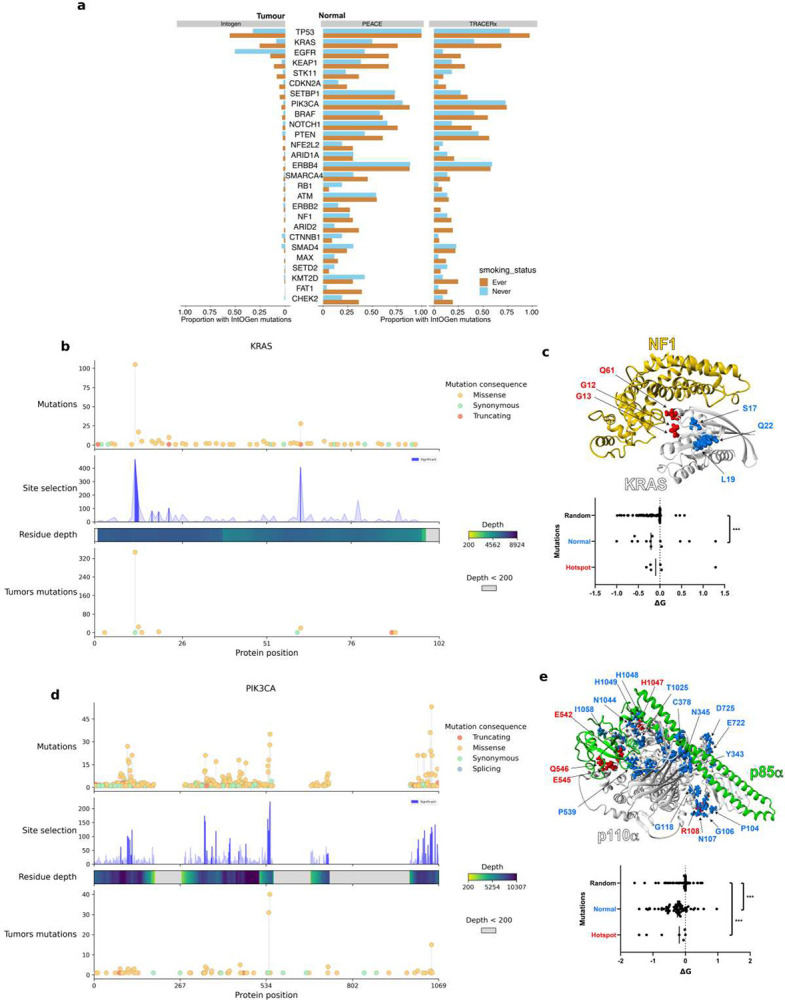
Smoking influences prevalence of site-specific mutations in lung cancer-associated genes. **a**, Bar graphs of the proportion of lung samples sequenced in the IntOGen database (NSCLC), PEACE cohort, and TRACERx cohort (left to right) harboring IntOGen mutations across genes stratified by smoking status. **b** and **d**, Plots highlighting site selection for mutations in *KRAS* (**b**) and *PIK3CA* (**d**): Lollipop plot of mutation frequency stratified by mutation consequence (top) and density plot of the level of site selection for each amino acid of the mutations observed in the PEACE and TRACERx cohorts. A lollipop plot of IntOGen mutations in lung cancer tumors (bottom) is shown for comparison. **c** and **e**, 3D structure (top) of the proteins (white) encoded by *KRAS* (**c**) and *PIK3CA* (**e**) with IntOGen cancer hotspot mutations (red), additional observed mutations significantly under selection (blue), and the regulatory subunits NF1 (yellow) for KRAS and p85α (green) for PIK3CA. Calculations (bottom) of the Gibbs free energy change (ΔG) of hotspot, normal, and random mutations in *KRAS* (**c**) and *PIK3CA* (**e**). Significance by one-way Anova. Difference between Random and Normal is only significant for KRAS when considering absolute values.

We next compared the proportion of samples with mutations in each gene stratifying all the cohorts between ever-smokers and never-smokers. We used the mutations detected in IntOGen NSCLC as a subset of likely-functionally relevant mutations. For some genes, such as *KRAS*, *TP53* and *EGFR*, we observed high frequencies of IntOGen mutations in both normal lung and in NSCLC, in both ever-smokers and never-smokers (Fig. 4a). In contrast, for most other genes, including *SETBP1, PIK3CA, BRAF*, *ERBB4* and *ATM*, we observed far more IntOGen mutations in the normal lung tissue while these mutations are much rarer in NSCLC. Thus, mutational patterns in normal tissue at the gene level only partially reflect the spectrum of mutations that characterize lung cancers.

When we compared mutational patterns within a gene, we observed further differences between normal lung and lung cancer. Lollipop and selection plots for mutations in *KRAS* revealed overlap between mutational patterns in normal lung with those in NSCLC, with the strongest selection observed in normal lung for the known hotspot mutations found in NSCLC (at residues G12 and Q61) (Fig. 4b). Still, a number of additional residues were significantly under selection (S17, Q22 and L19; dark blue in site selection plot in Fig. 4b) and a broader pattern of selection for mutations extended across the protein coding sequences of *KRAS*.

Modeling both hotspot (red; top 5 mutations observed in NSCLC) and other residues significantly under selection (blue) onto the structure of the KRAS protein (with PDB accession 6V65)^41^ revealed clustering of these non-hotspot residues (S17, Q22 and L19) near the interface with the GTPase-activating protein NF1, a key negative regulator of RAS activity (Fig. 4c). Using mCSM-PPI2^42^, which calculates predicted changes in the energetics of the association between KRAS and NF1 due to mutations, we calculated the predicted changes in ΔG (Gibbs free energy of binding) for this interaction. The ΔG is an estimate of the thermodynamic affinity between a ligand and a target protein, and as defined by mCSM-PPI2, lower ΔG indicates weaker affinity. While estimated changes by mCSM-PPI2 were not significant in ΔG for either hotspot or normal tissue selected mutations, there was a significant (p<0.0001; one-way Anova) change in the *absolute* values of ΔG for normal tissue mutations relative to random mutations in the same region (based on mutational expectations). Thus, the somatic mutations are more likely to change energetics of the association with NF1 compared to random mutations (although the changes go in both directions). The outlier with positive ΔG is Q61H, which we predict increases the interaction with key arginine residues (R1276, R1391) in NF1, leading to a slower off-rate with NF1 and disrupted GTPase activity (through an unknown mechanism).

*PIK3CA* exhibited substantially more protein-altering mutations in normal lung relative to NSCLC (Fig. 4a). Selection for mutations in *PIK3CA* were distributed across the gene for normal lung (Fig. 4d), contrasting with NSCLC where more than 80% of mutations in *PIK3CA* are in five residues (R108, E542, E545, E546, H1047). Mapping the codons that are significantly under selection in normal lung onto the structure of the *PIK3CA*-encoded p110α catalytic subunit of phosphatidylinositol 3-kinase (PI3K; PDB accession 7PG5)^43^ bound to the regulatory p85α subunit reveals that many of these residues (shown in blue) are clustered near the interface with p85α, similarly to the known hotspot mutations in NSCLC (shown in red) (Fig. 4e). Note also the clustering of mutations in the structure, consistent with analyses in **Extended Data Fig. 5a-c**. All of the five hotspot residues for NSCLC were also significantly under positive selection in normal lung except R108. Importantly, when using mCSM-PPI2^42^ to calculate predicted changes in the energetics of the association between p110α and p85α due to mutations, both the known hotspot mutations and the mutations in *PIK3CA* selected for in normal lung demonstrate a significant decrease in ΔG (p<0.001; consistent with reduced affinity) relative to randomly generated mutations. The E542K and E545K mutations in the helical domain and the H1047R mutation in the kinase domain have been shown to alter association with p85α^44^, leading to more constitutive PI3K activity. Thus, mutations in PIK3CA selected for in normal lung appear to perturb this association with the regulatory p85α subunit in a manner analogous to the cancer hotspot mutations. In all, these results reveal that selected mutations in normal lung, even when not contributing to cancer, are likely altering key interactions with regulatory protein subunits in a manner similar to that observed for hotspot mutations.

Finally, site specific selection for specific mutations in *BRAF*, *EGFR* and *TP53* overlap with but were distinct from hotspot mutations in NSCLC (**Extended Data Fig. 9**). Thus, across all five of the prioritized genes, selection across codons was substantially more distributed relative to the patterns observed in NSCLC.

### The impact of smoking on cancer hotspot mutations in genes under selection in normal lung tissue

**Figure 5.**
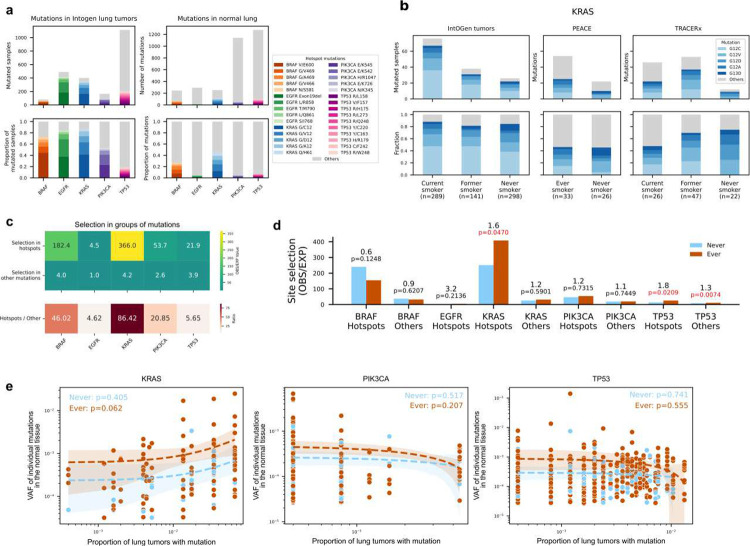
Smoking increases the prevalence of lung cancer hotspot mutations in normal lung. **a**, Bar plots highlighting the prevalence of hotspot mutations in genes under positive selection: On the left, the number (top) and proportion (bottom) of IntoGen lung tumor samples harboring non-synonymous mutations in the IntOGen database. On the right, the number (top) and proportion (bottom) of observed non-synonymous mutations in normal tissue from the PEACE and TRACERx cohorts. The colored bars represent hotspot mutations in the designated gene. **b**, Stacked bar plots stratified by smoking status of the number (top) and proportion (bottom) of samples harboring BoostDM *KRAS* driver mutations with hotspot residues graphed for the lung tumors in the IntOGen database (left), normal lung in the PEACE cohort (middle), and normal lung in TRACERx cohort (right). **c**, Heatmap representations of the site selection values for hotspot and non-hotspot mutations in the five prioritized genes in the normal lung (top) and of a ratio of these calculated values (bottom). **d**, Bar plots of calculated site selection stratified by smoking status for hotspot and non-hotspot mutations in five positively selected genes. Significant site selection is observed for hotspot and other mutations for all five genes (except non-hotspot/other mutations for *EGFR*, which are not graphed). The fold difference between the site selections in the smoking statuses is provided above the bars, and the p-value of a likelihood ratio test comparing Poisson rates between smokers and non-smokers is shown below. p-values in red are significant. **e**, Scatter plots of the variant allele frequencies (VAF) of *KRAS* (left), *PIK3CA* (middle), and *TP53* (right) mutations versus its proportion in IntOGen lung tumors, stratified by smoking status (p-values from linear regression); p-values of linear regression between the two variables are shown for each group of smokers and non-smokers.

For *TP53*, *KRAS*, *EGFR*, *PIK3CA* and *BRAF*, we quantified the prevalence of the top 5 (top 10 for *TP53*) most frequent NSCLC “hotspot” mutations observed in the IntOGen database (Fig. 5a). These hotspot mutations represent a substantially smaller fraction of total non-synonymous mutations in each of these genes in normal lung tissue than in IntOGen NSCLC. Stratifying these observations by smoking status, and limiting comparisons to IntOGen designated mutations, revealed that non-hotspot IntOGen mutations are overrepresented in normal lung tissue relative to NSCLC for all five genes (Fig. 5b and **Extended Data Fig. 10**). We observed fewer G12C mutations in *KRAS* in never-smokers, as expected given that this mutation is strongly associated with tobacco-mediated mutagenesis^45^.

We next asked if selection of hotspot mutations in normal tissues is also higher compared to other mutations in these same genes. Measuring selection as the ratio between observed and expected mutations under neutral mutagenesis in the two groups of mutations, we confirmed that indeed this was the case (Fig. 5c). This difference was particularly high for *KRAS*, *BRAF* and *PIK3CA*. Notably, selection for non-hotspot mutations is still significant for all genes except *EGFR*. Calculating site selection after stratifying the samples by smoking status (Fig. 5d), we observed significantly stronger selection in ever-smokers relative to never-smokers for *KRAS* hotspot mutations and in *TP53* for both hotspot and non-hotspot mutations, the two most commonly mutated genes in smoking-associated lung cancers^35^.

Notably, we observed no significant positive association between the frequency of IntOGen mutations in *KRAS*, *PIK3CA* and *TP53* in lung cancers with their detected frequencies in normal lung (Fig. 5e). Thus, clones with mutations that are rare in lung cancers have comparable sizes in normal lung tissue as clones with mutations that are common (hotspots) in lung cancers.

### Multiregional sampling reveals intra-individual convergent evolution

**Figure 6.**
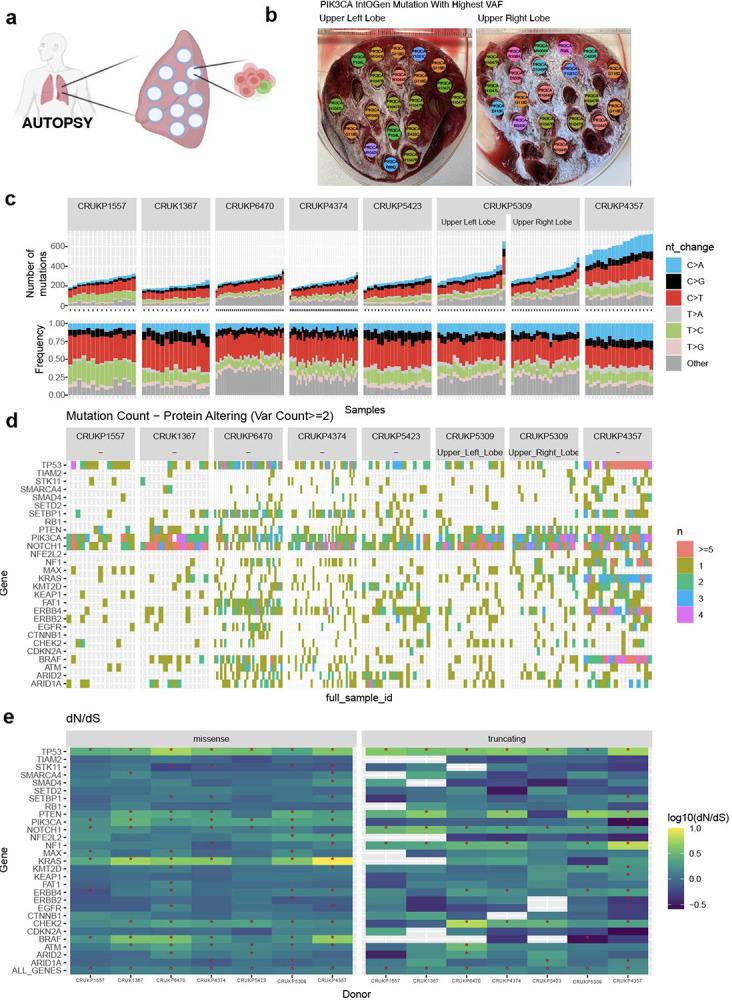
Multiregional sampling reveals convergent mutational selection at the gene level. **a**, Depiction of the acquisition of lung punches in the multi-region cohort (left). **b**, A representation of *PIK3CA* IntOGen mutations throughout the lung punches in the left and right lobes of participant CRUKP5309 (right). The mutation with the highest VAF in each sample is indicated. **c**, Counts (top) and proportion of mutational signatures (bottom) of all mutations. **d)** Heatmap of the counts of protein altering mutations stratified by gene (bottom) in the multi-region lung samples across seven participants. **e**, Heatmap of dN/dS calculations of missense (left) and truncating (right) mutations in each gene. The red asterisk indicates significant positive selection as defined by omega.

The PEACE, TRACERx and BDRE cohorts enabled us to investigate lung clonal expansions and how they are impacted by smoking from single samples across many individuals. We wondered how much a single sample gives a clear representation of the mutation landscape across the lung in an individual. To address this gap, we acquired 15 to 44 tissue punches across each lung of seven individuals, as depicted in Fig. 6a. As shown in these representative lung sections for *PIK3CA* mutations across two lung lobes within participant CRUKP5309 (Fig. 6b; **see Extended Data Fig. 11 for *EGFR***), most regions showed IntOGen-designated *PIK3CA* mutations, but the identity of the mutation with the highest VAF varied across regions within the same patient. These results are consistent with selection for enhanced PI3K activity across the lung, with different PIK3CA mutations providing this adaptation in different regions.

The frequency and type of mutations are shown in Fig. 6c, with greater total number of mutations and C>A (G>T) transitions seen in the two individuals who smoked (CRUKP5309 and CRUKP4357). In focusing on protein-altering mutations with at least two unique molecular identifiers (to enrich for clonally-expanded mutations) (Fig. 6d), we observed different mutation patterns in individuals across genes. *TP53, PIK3CA*, and *NOTCH1* were consistently mutated across the lung in all individuals (Fig. 6d). However, some genes exhibited individual-specific mutation patterns such as SETBP1, *ERBB4*, *FAT1* and *BRAF* in CRUKP6470, with high mutation counts across many of the genes for CRUKP4357. In general, mutations in *SETBP1, NF1, FAT1, ERBB4, EGFR, BRAF, ATM, ARID2* and *ARID1A* exhibited highly individual-specific mutation patterns.

To determine if patterns of selection are also evident across punches within these individuals, we calculated dN/dS for missense & truncating mutations and quantified indel density for each gene. Missense mutations in *TP53, PIK3CA*, *KRAS*, *BRAF, ATM* and *CHEK2* were under positive selection across almost all individuals (Fig. 6e). Truncating mutations in *TP53*, *NOTCH1* and *NF1* also exhibited positive selection in almost all individuals. Other genes, such as *SMARCA4*, *SETBP1*, *ERBB4* and *EGFR* exhibited individual-specific selection. Thus, each individual has a different spectrum of genes exhibiting positive selection, suggesting that each person has a distinguishable lung adaptive landscape. Additional analyses of sum of VAF for protein-altering missense and indel mutations, or counts of IntOGen mutations, similarly revealed individual-specific patterns of mutations (**Extended Data Fig. 12**). Thus, somatic evolution is relatively reproducible and predictable in a given person’s lung.

## DISCUSSION:

Cigarette smoking is well known to negatively impact lung function and to substantially increase the risks for lung diseases including cancers^46^. However, how smoking impacts the clonal mutational landscape and consequently increases risks for oncogenesis and other diseases in the lung have not been thoroughly investigated. Here, we conducted DuplexSeq to analyze rare somatic mutations in histologically normal, cancer-free lung tissue across three cohorts with participants of varying smoking histories. While each cohort study has its limitations, such as treatment for many patients in PEACE, proximity of normal lung sampling to cancer in TRACERx, and the paucity of never-smokers in BDRE, these three cohorts have complementary characteristics that provide confidence in our overall conclusions regarding mutational patterns and selection in the normal lung.

Consistently across all cohorts, we observed an increase in the mutation burden with smoking, reflecting some combination of increased mutagenesis and enhanced positive selection. Importantly, we observe clear presence of and selection for cancer-associated mutations in both never and ever smokers, including for hotspot mutations in *KRAS*, *TP53* and *EGFR* frequently found in lung cancers. Critically, smoking substantially influences *which* cancer driver mutations are selected and expand, and the *strength* of this selection. Smoking alters the lung tissue landscape to increase the fitness of cells possessing mutations with the highest prevalence in lung cancers, including hotspot mutations in *KRAS* and *TP53*. It is important to note that most mutations under selection will rarely be associated with a lung cancer (as discussed for other tissues^16^). In normal lung, we observe selection in cancer-associated genes outside of the traditional hotspots, which is particularly evident for *PIK3CA*, *BRAF* and *EGFR*. Through protein modeling, we show that many of these selected, non-hotspot mutations are predicted to impact protein function in a fashion analogous to the hotspot mutations.

So why do these other mutations rarely associate with lung cancers? Cancer evolution may require a “just right” level of a protein activity. Alternatively, different mutations could have different impacts on pleiotropic activities of a protein with selection acting on one activity but tumor evolution requiring a particular combination. Additionally, we observe no significant correlation in the VAF of observed mutations and their prevalence in lung cancers. Previous studies of mutational patterns in normal tissue have observed a similar disconnect at the gene level, with positive selection for mutations in genes such as *NOTCH1* leading to clonal expansions in the normal tissue that far outpace their frequency in associated cancers (reviewed in^16,23,47^). These results suggest that only a small fraction of mutations that are adaptive within normal tissue are likely to contribute to a cancer, and that even cells with cancer-associated hotspot mutations in normal tissues are very unlikely to contribute to a malignancy. Context matters. Additional cell intrinsic (mutational and epigenetic) and extrinsic (e.g. inflammatory) changes must act upon mutational landscapes engendered by time and exposures over life to enable cancer progression from a rare clone. Future studies are needed to better predict the odds that cells with cancer driver mutations will contribute to cancer evolution, the factors (from exposures to genetics to aging) that change these odds, and how these mutation-driven expansions alter tissue functionality and the risks of non-malignant diseases. Understanding the causes and consequences of mutation-driven clonal expansions in normal tissues should also enable the development of interventions that promote clonal patterns with low disease risk, such as by favoring more benign mutation-driven clonal expansions that effectively compete against more malignant ones^48,49^.

Multiple studies have shown how factors such as aging and therapies can influence clonal selection in normal tissues^19–21,28^ (reviewed in^16,23,47^). While most of our subjects were older, impeding our ability to determine the influence of age on the strength of selection, we do calculate clear alterations in selection for mutations in specific genes corresponding to treatments received. It is notable that different therapies exert very different effects on selection for different genes. Importantly, we also observe how smoking can both alter which genes exhibit altered selection following different therapies and generally obscure signals of selection mediated by therapies. In never-smokers, however, where baseline mutation burden and altered selection are lower, treatment-induced damage or immune modulation may create a permissive environment for selection of pre-existing driver clones. While such altered selection is easier to envision for cytotoxic therapies, it is less clear why immunotherapy, which should enhance recognition of mutation-created neoantigens, would increase positive selection for particular gene mutations (unless competing non-mutant cells are somehow impeded). Overall, these results have important implications for understanding the evolution of secondary cancers – different anti-cancer therapies, influenced by smoking, will substantially alter mutational landscapes in normal tissues that could later spawn new cancer evolution.

Finally, through our multi-region analyses, we observe similar mutational and selection patterns across different regions within each individual. These individual-specific patterns indicate that somatic evolution converges on particular adaptive solutions within an individual, often by selecting for different mutations in the same gene across the lung. This convergence likely reflects an amalgamation of many factors including underlying genetics, lifestyles, and lifetime exposures (including tobacco, particulates, infections, and therapies) driving convergent somatic evolution. Understanding the adaptive landscape of an individual’s normal tissue, and how exposures and lifestyles alter this landscape, could enable us to anticipate and perhaps counteract somatic evolution that can lead to cancer or other diseases.

## METHODS:

### Sample procurement

This project utilized samples obtained from the national pan-cancer research autopsy program (PEACE, NCT03004755), the prospective, longitudinal cohort study (TRACERx) of NSCLC (NCT01888601), and the Biomarkers and Dysplastic Respiratory Epithelium (BDRE) study (NCT00900419). For the PEACE study, 66 healthy lung tissue samples were collected at post-mortem. Each piece of collected tissue was immediately bisected, and one half was snap-frozen and the other was fixed and made into a formalin-fixed paraffin-embedded (FFPE) block. The H&E section of each block underwent pathology review to identify tissue samples that were histologically normal for DNA extraction. DNA was then extracted from a directly adjacent frozen healthy tissue sample. Seven participants had unknown smoking statuses, so their samples were not used for downstream analyses. 42 of the remaining 59 participants received therapy for their malignancies. Only 16 of the 59 samples were from people who had lung cancer, and they did not show notable differences in mutational profiles (including measures of selection) from samples from the other PEACE subjects who had cancer at distant sites.

For the TRACERx study, 95 non-cancerous lung tissue were obtained at surgery and collected distally from the primary tumor tissue (at least 2 cm apart). All tissue was initially snap-frozen and then a portion fixed and made into a FFPE block. A hematoxylin and eosin (H&E) section of each block underwent pathology review to identify tissue samples that were histologically normal for DNA extraction. DNA was extracted from frozen tissue. At the time of resection, none of the subjects had received therapy for lung cancer, and only seven of them had received therapy for a previous cancer.

All aforementioned H&E slides from tissues underwent central pathology review. In particular, to exclude the possibility of contamination with tumor cells, thoracic pathologists confirmed that all healthy lung tissue samples did not contain any indication of tumor tissue or morphologically defined, pre-invasive disease.

Participants in the BDRE study (NCT00900419) consisted of individuals recommended for a low-dose computed tomography (LDCT) scan based on age, smoking history, or other indications. If a suspicious nodule was detected by CT scan, a navigational bronchoscopy was indicated. The nodule site was sampled for accurate diagnosis. For each patient, a brushing from a remote site in a contralateral lobe was also taken for research as a representative sample of non-cancerous tissue, frozen, and then processed for DNA extraction. The absence of nodules or masses detected by chest CT scans was indicative of the non-tumor nature of these contralateral samples. Each procedure was performed under fluoroscopic guidance, with the brush advanced from the sheath only after documentation that the working channel was in the peripheral airways.

### Duplex-seq

Genomic DNA was extracted from samples using a Qiagen DNeasy Blood and Tissue kit according to the manufacturer’s instructions. Duplex libraries were prepared using a commercially available kit from TwinStrand Biosciences (CKD-00042 panel 000323). Custom probes were designed for targeted capture hotspot regions in 31 genes recurrently mutated in lung cancer, and *TIAM2*, which was used as an internal reference gene (**Extended Data Table 1**). We excluded *KMT2C* from downstream analyses, as the presence of numerous pseudogenes makes it difficult to identify true somatic mutations^50^. We also excluded intronic regions for *ALK*, *RET* and *ROS1*, which were originally intended for the detection of translocations. Thus, results are presented for 27 cancer genes and *TIAM2*.

By independently capturing and sequencing the two strands of DNA for selected genomic regions, combined with the use of a common unique molecular identifier for both strands, Duplex-seq enables the detection of rare mutations^51^ with a sensitivity of less than 1 in 10^4^ (given our sequencing depth). After shearing and capturing of gDNA spanning the panel, primers were ligated so that the two strands of DNA for each segment were uniquely labelled and matched with its opposing strand. These strands were then amplified, and libraries were sequenced on a NovaSeq 6000 sequencing system (Illumina), and sequencing data were processed with deepUMIcaller to obtain all mutation calls with annotated mutation filters and information of the duplex depth per site.

For PEACE, TRACERx, and multi-region samples, we used an input of 400ng DNA into the enzymatic fragmentation step for shearing. We obtained an average of approximately 250 million raw reads with this DNA quantity. For BDRE bronchoscopy samples, we used input DNA quantities of 250ng for contralateral site samples and 100ng for the involved site samples (where the initial suspected cancer lesion was detected through CT scan) into the end repair/A-tailing reaction after shearing by sonication. We acquired approximately 200 million and 100 million raw reads for the contralateral and involved site samples, respectively.

### IntOGen tumor mutations data

Database tumor data was obtained from the cohort available in IntOGen^35^ (intogen.org). When restricting the analysis to lung tumors, we used information from 2,503 tumors belonging to non-small cell lung cancer (NSCLC) cohorts, including both lung adenocarcinoma and squamous cell carcinoma cases. The clinical information, in particular smoking status, was not available for all cases, and this restricted the size of the cohorts for stratified analysis. Sample sizes of the different subgroups were indicated in the figures or figure legends.

### Positive selection

To provide a sense of both mutation occurrence and their expansion, we calculated sums of VAFs for the cohorts. This metric is simply the addition of the calculated VAFs of nonsynonymous, coding mutations. When calculated across all genes or by a specific gene, this sum was normalized by the length of the entire panel or the gene in question, respectively. Assuming heterozygosity and that the mutations do not co-occur in the same cell, this measure can provide insight as to the percentage of cells harboring a gene or pathway’s mutations.

We used omega^21^ for the dN/dS estimations of missense and truncating mutations, both at the level of cohort and for the group comparisons. At the cohort level, genes with a p-value < 0.05 were defined as significantly selected for missense or truncating mutations, respectively. Truncating mutations include nonsense SNVs and SNVs leading to essential splice consequence types defined by Ensembl VEP. For the comparisons between smokers and non-smokers, the same approach was used but focusing only on those genes with significant selection at the cohort level and with enough synonymous mutations observed in that gene-group of samples combination to obtain a stable estimation of the background mutation density. For the comparisons of the BDRE cohort, the small number of synonymous mutations prevented this same approach, and we leveraged the cohort synonymous mutation density to inform the estimation of the expected neutral mutations for each gene.

For the driver gene discovery analysis, we also run OncodriveFML^38^, to measure selection in the targeted genes based on functional impact bias, and Oncodrive3D^37^ to measure the 3D clustering of missense mutations within a protein.

We used the mutabilities option of omega to generate the expected number of mutations per specific site and sample (or group of samples). These were then used for comparing with the observed number of mutations and to obtain an estimate of the selection per site, following the implementation in deepCSA (https://github.com/bbglab/deepCSA).

### Hotspots definition

The definition of tumor hotspots for *KRAS, TP53, BRAF, PIK3CA* and *EGFR* was done based on the most common mutations in these 5 genes when using all NSCLC included in IntOGen. For all genes we used the top 5 mutations, except for *TP53* where we increased the list to the top 10 mutations, detected in the regions of these genes that overlapped with the ones we sequenced in the normal tissues to ensure that all comparisons between the two sets of mutations are done allowing for the same mutations.

EGFR exon 19 deletion was defined as any in-frame insertion or deletion between protein positions 729 and 761, and any in-frame insertion between 762 and 823. All the mutations matching these criteria were grouped together as if they were the same mutation.

### Signature analysis

We extracted mutational signatures de novo with a Bayesian hierarchical Dirichlet process using an adapted version of the HDP_sigExtraction pipeline (https://github.com/McGranahanLab/HDP_sigExtraction) and the R-package hdp developed by N. Roberts (https://github.com/nicolaroberts/hdp). The HDP_sigExtraction pipeline was run with the default parameters with no previous signatures assigned. We also used SigProfilerExtractor, a non-negative matrix factorization tool to extract mutational signatures. This was also run with default parameters, and the suggested solution was used for the comparison with HDP results. The accuracy of the extracted signatures and their reconstruction using COSMIC signatures is heavily affected by the limitations imposed by the small number of mutations per sample and the different representation of all trinucleotides in the targeted region. For this reason, the extracted components SBS_A and SBS_B are used for the downstream analysis.

### Statistical analysis

For the group comparisons of numerical values (e.g. mutation density and sum of VAF across smoking status or proportion of signature mutations), the student’s T-test or Mann-Whitney U (also called Wilcoxon Ranked Sum) test is used. If the data are confirmed to be normal for both categories, the T-test is used. Otherwise, the Mann-Whitney U test is conducted. All tests were two-sided unless specifically noted.

Comparisons of dN/dS values between smoking groups were done using a Fisher’s exact test on the proportion of driver and non-driver mutations in each smoking/cancer group. The numbers of driver and passenger mutations were computed using the proportion of excess mutations. Representing a dN/dS estimate as *ω* the numbers are computed as follows:

$$\text{Driver}\;\text{mutations}=\text{Observed}\;\text{mutations}\cdot \frac{\left(\omega -1\right)}{\omega }$$

Expectedmutations=Observedmutations−Drivermutations

### Mutation filtering

After extracting mutations from FASTQ files, we conducted postprocessing to filter out mutations with questionable read quality using various criteria. Across the single-region cohorts, there is a total of 46,929 mutations. We removed mutations with a duplex depth less than 500 (681 mutations), mutations on reads with 20 or more undetermined nucleotides (0 mutations), mutations in N-rich regions (1602 mutations), mutations determined to be single nucleotide polymorphisms (1096 mutations), mutations on reads with low mappability (0 mutations), mutations missing from pileup output from associated BAM file (40 mutations), and mutations with discordant VAF calculations upon incorporation of single reads (0 mutations).

Then, we applied filters to remove other likely germline mutations, artifactual mutations, and mutations that we concluded were due to contamination. Only 28 mutations were observed with VAFs (calculated by either duplex reads, total duplex and single reads, or VarDict) above 30% and were crosschecked in the gnomAD database. They were eliminated to ensure we did not have germline mutations or polymorphisms in our somatic mutation analyses. For artifacts, we removed an array of insertions localized in a 10-bp span of *RB1*’s intron 13 that was observed 400 times. Additionally, *ROS1, RET*, and *ALK* were initially added to our panel in hopes of potentially detecting translocations in intronic regions. This proved to be an inappropriate platform for such an investigation and led to an influx of artifactual intronic SNVs and indels. We removed these three genes and their mutations from our downstream analysis. Also, *KMT2C* is a gene with many pseudogenes that reduced confidence in proper read alignment^50^. We also removed this gene from downstream analysis.

PIK3CA mutations were called and filtered independently. The hotspots at protein positions 542 and 545 are not covered when applying stringent AS-XS mappability thresholds; to overcome the loss of these mutations we reduced the AS-XS threshold at 10. Apart from this change the rest of filters were applied equally.

For each cohort, we also sequenced matching samples to eliminate potential mutations due to tumor and blood contamination. In the PEACE cohort, tumor samples from 33 of the 59 participants were sequenced with MiSeq in which 671 mutations were observed within the regions targeted in this study. 19 of these mutations were present across 9 patient-matched normal lung samples for our studies. These mutations had VAFs below 1% and were likely to be inconsequential to downstream analysis. Therefore, these mutations were retained. In the TRACERx cohort, patient-matched tumor samples were analyzed. In a single sample of the normal tissue duplex sequencing, a high-VAF *STK11* mutation was detected and coincided with one of the driver mutations of the matched tumor. We discarded this sample given this evidence of tumor contamination in the normal tissue. For the BDRE cohort, we observed 3 mutations (2 in *NOTCH1* and 1 in *FAT1*) that were observed in both the involved and contralateral bronchoscopy brushing sites. However, all mutations had VAFs no greater that 0.5% on either side, suggesting that it is unlikely that these clones came from circulating DNA or the blood. We retained these mutations in contralateral mutational analyses.

Likewise, there is a total of 72,663 multi-region mutations. We removed mutations with a duplex depth less than 500 (285 mutations), mutations on reads with 20 or more undetermined nucleotides (0 mutations), mutations in N-rich regions (1424 mutations), mutations determined to be single nucleotide polymorphisms (353 mutations), mutations on reads with low mappability (0 mutations), mutations missing from pileup output from associated BAM file (153 mutations), and mutations with discordant VAF calculations upon incorporation of single reads (0 mutations).

In the samples from our multi-region study, 18 mutations were observed in over 80% of the regions within a particular individual, accounting for 352 unique mutations. Although these mutations were largely exclusive to a particular person and they were not observed in tumor samples, we eliminated them due to uncertainty that they could be from the blood. It is worth noting that there is still evidence of convergent evolution despite this elimination. Additionally, there were two *TP53* mutations observed in each tumor sample from patients CRUKP6470 and CRUKP1557. Due to their presence across multiple regions, we eliminated these mutations from these two samples.

With this set of mutations, the major downstream the analysis were run using deepCSA (https://github.com/bbglab/deepCSA).

Regarding the tumor mutation data that was obtained for the above analyses, for TRACERx samples, this was obtained from the TRACERx study^52^. For the PEACE sample, we obtained data from the PEACE study^32^ where it was available. Where it was unavailable, we performed MiSeq on the tumor closest to where the normal lung tissue was harvested. Tumor MiSeq amplicon panel data from FASTQ files were processed through the nf-core Sarek pipeline run in exome mode. Because matched normal samples were unavailable, a tumor-only caller configuration was applied alongside a panel of normals, a dbSNP database, known indels, and the gnomAD common population alleles to filter out germline single nucleotide polymorphisms (SNPs) and sequencing artifacts. Specifically, Mutect2 was utilized as the somatic variant caller to identify tumor-specific single nucleotide variants (SNVs) and indels through a Bayesian model and statistical filters. Following variant calling, the Ensembl Variant Effect Predictor (VEP) tool was employed to annotate the genetic variants and predict their functional biological consequences.

### Excluded samples

BDRE sample 154 was excluded due to an unexpectedly high number of mutations and a mutational profile highly similar to potentially artifactual mutations. See figure below.


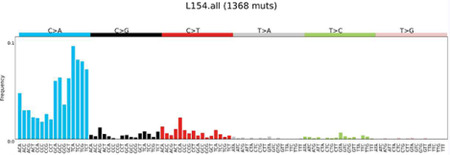


### Limitations:

This study involves deep mutational analyses on numerous samples across complementary cohorts. However, there are some limitations that merit attention. Although there are not notable distinctions in confounding characteristics (i.e. age, sex, BMI or therapy) across smoking status (**Extended Data Figure 2**), we cannot definitively rule out the potential role for these or other factors in influencing our results. The BDRE cohort is male-dominated and does not have many never-smoker participants, restricting sex comparisons in bronchoscopic brushings. Furthermore, the nature of sampling restricts proper comparisons between cohorts. Based on previous work where imaging mass cytometry was conducted on normal lung tissue, we estimate that the lung samples from the PEACE and TRACERx cohorts contain approximately 26% epithelial cells^33^. On the other hand, for BDRE, brushing samples from the uninvolved contralateral lung are enriched for bronchial epithelial cells (>89%)^29,53,54^. Additionally, our targeted sequencing panel emphasizes genomic locations where lung cancer-associated mutations have been previously found, limiting the genes and gene regions analyzed for mutations and our ability to build a more stable estimation of background mutations for determination of positive selection. Furthermore, without dedicated loci known in clonal hematopoiesis, we cannot rule out contamination from CHIP clones in blood in the samples. Still, such contamination would be expected to be evident in matched samples from the same individual (such as for involved and contralateral sites in BDRE, or across regions in the multiregional sampling), and we detected very few such mutations (see above). Thus, if contaminating mutations from circulation exist, such mutations do not appear to contribute more than a very small fraction of the mutations detected in our samples.

## Supplementary Material

Supplementary Files

This is a list of supplementary files associated with this preprint. Click to download.

- ExtendedDataFiguresXTables.docx
- extendedDataTable2metaData.xlsx
- nrreportingsummaryEvansEtAl.pdf
- extendedDataTable1panelWithGenes.xlsx

## Data Availability

Duplex sequencing data will be made available at the European Genome-Phenome Archive (EGA) upon publication of the study. These will be available for academic, noncommercial research purposes subject to a review of a project proposal by the corresponding data access committee (PEACE, TRACERx, or BDRE).

## CODE AVAILABILITY

All the analysis can be reproduced from the raw duplex data by running deepUMIcaller and deepCSA. Code for the analysis and figures will be made available on Zenodo upon publication of the manuscript (currently on GitHub).
